# Scottish Medical Service Emergency Committee

**Published:** 1915-07-10

**Authors:** 


### The Hospital

The Workers' Newspaper of Administrative Medicine and Institutional Life, Administration, National Insurance and Health.

No. 1517, Vol. LVIII. SATURDAY, JULY 10, 1915. PRICE ONE PENNY

### SCOTTISH MEDICAL SERVICE EMERGENCY COMMITTEE.

This Committee was constituted in August 1914 for the purpose of dealing with the problems which called for solution at the beginning of the war. It includes representatives of all the Universities, of the Colleges of Physicians and Surgeons, and of those engaged in general practice. In its earlier days it undertook effectively much responsible work, and the recent call of the Director-General has created for the Committee a further opportunity. Both the War Office and the medical profession are more than content that it should continue its activities and that it should be the official channel of communication between the military authorities and the medical profession in Scotland. Of course it has no power of control, but it is prepared to advise, to suggest, and to direct, and its representative character and carefully-gathered experience guarantee its full equipment for these duties.

From a circular recently issued by the Committee we are able to learn something both of the position in Scotland and of the probable wants of the Military Medical Service. An estimate has been made that the further number of medical practitioners which Scotland may be expected to supply is 400. Towards these the graduates and licentiates of the present month are expected to contribute 100. The balance of 300 may be called for in three successive series—namely, in August, September, and October. All these figures, be it understood, refer to men willing to give whole-time service and to accept a commission in the R.A.M.C., with the pay of 24s. a day, a ration allowance of 1s. 9d. per day, £30 to be granted for outfit, and a gratuity of £60 at the termination of service provided this has been satisfactory and has extended over not less than twelve months; in the event of the enlistment being for six months only the retiring gratuity is reduced to £15. Of great importance is this question of length of service. Previously it was understood that the acceptance of a commission meant an undertaking to serve for three years or for the duration of the war, whichever of these two dates meant the shorter period. But now we have it definitely set forth that men under forty may volunteer for twelve months and that during this period they are liable to serve either at home or abroad, while men over forty may choose a term either of six or twelve months, with an undertaking that while they may be sent to duties either at home or in such stations as Egypt, Malta, and Gibraltar, they will not be sent to France. As these statements are authoritatively issued by the Committee they must be accepted as the present policy of the War Office, and obviously their importance cannot be exaggerated. The fear of an indefinite and probably prolonged absence from practice has debarred many men from offering the help they would otherwise gladly have rendered, and now it is known that the War Office will accept short and definitely fixed periods the number of possible volunteers is considerably increased. Doubtless what is true for Scotland is true for other parts of the kingdom, though no Committee save the Scottish one appears to have persuaded the War Office to this new modification.

The task, then, which lies before the Committee is to raise in Scotland during the next three months at least 300 medical practitioners willing to give whole-time service to His Majesty's Forces. The methods by which it is proposed to accomplish this task have about them the quality of business-like thoroughness. Steps have been taken to secure an accurate and precise muster roll of the whole profession in Scotland. In addition, the country is to be divided into certain local administrative units, each one of which is to be in charge of a War Committee to be elected by the local profession. When the fresh drafts are called for the Central Committee will intimate to each War Committee the exact number of medical practitioners which the district is called upon to furnish; and of course it will be known whether any given district has or has not fully realised its opportunity and discharged its duty. Instead, therefore, of a vague appeal for an indefinite number of volunteers the call will come in the shape of precise figures, and there will be associated with it the force and influence of local patriotism. All honour will be due to each district which answers worthily the appeal, while a defaulting district will be reduced to the excuses for which a national emergency has but an impatient ear. Necessarily for those who remain behind is the care of the interests of their more youthful and more fortunate colleagues. To this point the Scottish Committee has given detailed attention. It has not trusted to a vague presentation of this responsibility, but has considered with care the various situations which may exist and how these can satisfactorily be met. Thus in its circular it discusses the adjustment of fees between the absent practitioner and his deputy as this requires recognition in the various classes of practice, and it urges very strongly that this adjustment shall be settled in advance and on a clear and business-like basis. Further, the Committee is prepared to consider any individual case which may be submitted to its judgment and to give advice, not only to the War Committees already described, but also to individuals who desire direction. In short, in addition to its general powers of co-ordination and direction it will act as a consultative organisation on all questions, individual or collective, which the present national situation presents for settlement. By its means every district in Scotland will learn the exact measure of its duty, and the War Office will know to what extent the demands of the situation can be satisfied. Regarding part-time service it is recognised that the arrangements for this can probably best be made locally and by those in actual touch with the situation of the various individual areas. The pressing question, as has repeatedly been urged in The Hospital, is the provision of medical officers for the new Armies. On such conditions as the Scottish Committee announces, the chance of answering this question satisfactorily has much improved. It means sacrifice, unselfishness, and loyalty, but these are hardly new claims to the medical profession.

